# Identification of late assembly domains of the human endogenous retrovirus-K(HML-2)

**DOI:** 10.1186/1742-4690-10-S1-P4

**Published:** 2013-09-19

**Authors:** Claudia Chudak, Nadine Beimforde, Maja George, Anja Zimmermann, Veronika Lausch, Kirsten Hanke, Norbert Bannert

**Affiliations:** 1 Division for HIV and other Retroviruses, Robert Koch Institute, Berlin, Germany

## Background

Late assembly (L)-domains are protein interaction motifs, whose dysfunction causes characteristic budding defects in enveloped viruses. Three different amino acid motifs, namely PT/SAP, PPXY and YPXnL have been shown to play a major role in the release of exogenous retroviruses. Although the L-domains of exogenous retroviruses have been studied comprehensively, little is known about these motifs in endogenous human retroviruses.

## Material and methods

A molecular clone of the human endogenous retrovirus K113 had been engineered to reverse the presumed non-synonymous postinsertional mutations in the major viral genes. Potential L-domains in the molecular clone expressing viral particles were inactivated and the release and budding phenotype analyzed by RT-assays and Western blot. Rescue experiments by Tsg101 and Alix overexpression and colocalisation studies were performed to substantiate the results.

## Results

We identified three functional L-domains of the virus, all located in the Gag p15 protein. A consensus PTAP tetrapeptide serves as the main L-domain for the virus and its inactivation reduces virus release in HEK 293T cells by over 80%. Electron microscopy of cells expressing the PTAP mutant revealed predominantly late budding structures and budding chains at the plasma membrane. The fact that this motif determines subcellular colocalization with Tsg101, an ESCRT-I complex protein known to bind to the tetrapeptide, supports its role as an L-domain. Moreover, two YPXnL motifs providing additional L-domain function were identified in the p15 protein. One is adjacent to the PTAP sequence and the other is in the p15 N-terminus. Mutations in either motif diminishes virus release and induces an L-domain phenotype while inactivation of all three L-domains results in a complete loss of particle release in HEK 293T cells. The flexibility of the virus in the use of L-domains for gaining access to the ESCRT machinery is demonstrated by overexpression of Tsg101 which rescues the release of the YPXnL mutants. Similarly, overexpression of Alix not only enhances release of the PTAP mutant by a factor of four but also the release of a triple mutant, indicating that additional cryptic YPXnL domains with a low affinity for Alix may be present. No L-domain activity is provided by the proline-rich peptides at the Gag C-terminus.

## Conclusions

Our data demonstrate that HERV-K(HML-2) release is predominantly mediated through a consensus PTAP motif and two auxiliary YPXnL motifs in the p15 protein of the Gag precursor.

